# Case Cured

**Published:** 1892-09

**Authors:** Alexander Villers

**Affiliations:** Dresden


### CASE CURED.

ALEXANDER VILLERS, M. D., DRESDEN.

Translated by A. McNeil, M. D., San Francisco.

A neurasthenic young lady consulted me for fatigue of the vocal organs in the upper register. There was a suspicion on my part of hysterical paralysis of the vocal cords. Repeated examination did not confirm this view and my treatment was in vain for ten days. I then had her sing the scales and vocal exercises to me. I immediately perceived that on account of deficient voice culture that she was compelled to strain her voice in singing the upper register. On consulting her teacher my opinion was corroborated.

I gave her Arnica$^{cc}$ a dose every day for three days, and requested her also to take a similar dose after each lesson.

In the five months which have elapsed there has been no new attack of fatigue of the voice.

I have had the pleasure of being consulted by a large number of vocal artists, and consequently I have had not a few cases of vocal fatigue. In the treatment of them I have gradually learned to differentiate. Arnica only cures in cases of over-exertion of a *deficiently* trained voice and in those quite rare ones where the modern use of the voice, which is in such bad taste of compelling a singer in opera to scream a *declaimed* musical passage above a full orchestra.

Argentum-nitricum is the remedy in exhaustion of a well-trained voice by using it too much or under unfavorable conditions, such as singing during menstruation.

Nux-vomica cures in all these cases of vocal fatigue, for which the striking expression of laziness of the mouth (*maulfaulheit*) is so expressive. The aversion to sing is only a part of the general aversion to corporeal exertion which so often indicates Nux-vomica.

[In the colds to which vocal artists are so subject, I have very frequently found Rhus-tox. to be *the* remedy.—Trans.]

In all the cases in which these three remedies are indicated, there are no [perceptible] abnormal states of the vocal cords nor any concomitants discernible.

Grauvogl's recommendation, *Lehr-buch Band*, II, page 268, to use Arnica oil in over-exertion of the voice, is not right. The higher potencies act surer and quicker, nor is the advice of a well-known homœopathic physician to gargle with a watery solution of Arnica any better. The gargling should always be avoided, and a couple of Arnica pellets, 30 or 200, if obtainable, act more certainly and without causing irritation.

From *Archiv für Homœopathie.*

